# An Efficient Self-Organized Detection System for Algae

**DOI:** 10.3390/s23031609

**Published:** 2023-02-01

**Authors:** Xingrui Gong, Chao Ma, Beili Sun, Junyi Zhang

**Affiliations:** 1 School of Computer Science and Engineering, Faculty of Innovation Engineering, Macau University of Science and Technology, Macau 999078, China; 2 Department of Engineering Science, Faculty of Innovation Engineering, Macau University of Science and Technology, Macau 999078, China; 3 Jiangsu Metabio Science & Technology Co., Ltd., Wuxi 214028, China; 4 Wuxi Key Laboratory of Biochips, Southeast University Wuxi Branch, Wuxi 214135, China; 5 Jiangsu Wuxi Environmental Monitoring Center, Wuxi 214121, China; 6 School of Environmental and Civil Engineering, Jiangnan University, Wuxi 214122, China

**Keywords:** algal blooms, algal detection, real-time detection, self-organized detection, Internet of Things (IoT), algal detection dataset

## Abstract

Algal blooms have seriously affected the production and life of people and real-time detection of algae in water samples is a powerful measure to prevent algal blooms. The traditional manual detection of algae with a microscope is extremely time-consuming. In recent years, although there have been many studies using deep learning to classify and detect algae, most of them have focused on the relatively simple task of algal classification. In addition, some existing algal detection studies not only use small datasets containing limited algal species, but also only prove that object detection algorithms can be applied to algal detection tasks. These studies cannot implement the real-time detection of algae and timely warning of algal blooms. Therefore, this paper proposes an efficient self-organized detection system for algae. Benefiting from this system, we propose an interactive method to generate the algal detection dataset containing 28,329 images, 562,512 bounding boxes and 54 genera. Then, based on this dataset, we not only explore and compare the performance of 10 different versions of state-of-the-art object detection algorithms for algal detection, but also tune the detection system we built to its optimum state. In practical application, the system not only has good algal detection results, but also can complete the scanning, photographing and detection of a 2 cm × 2 cm, 0.1 mL algal slide specimen within five minutes (the resolution is 0.25886 μm/pixel); such a task requires a well-trained algal expert to work continuously for more than three hours. The efficient algal self-organized detection system we built makes it possible to detect algae in real time. In the future, with the help of IoT, we can use various smart sensors, actuators and intelligent controllers to achieve real-time collection and wireless transmission of algal data, use the efficient algal self-organized detection system we built to implement real-time algal detection and upload the detection results to the cloud to realize timely warning of algal blooms.

## 1. Introduction

Algal blooms such as red tide [1,2] and cyanobacterial blooms [3] are occurring more and more frequently around the world and they not only devastate ecosystems but also seriously damage human health. For example, the blooms of dinoflagellate karenia brevis are thought to be capable of killing large numbers of fish and causing significant economic losses [4]. In 2005, the bloom of karenia in the Gulf of Mexico resulted in more than a month of benthic mortality [5]. In 2007, a cyanobacteria bloom in Wuxi, Jiangsu Province, caused a drinking water crisis for 2 million people [6], and a large cyanobacteria bloom took place in the western part of Lake Erie, Ohio, which cut off water supplies to over 500,000 people in 2014 [7]. Therefore, real-time monitoring of algae is necessary to prepare for and even prevent algal blooms before they occur, and how to quickly detect algae from acquired sample images is the core issue of real-time algal monitoring. The traditional manual detection of algae with a microscope is extremely time-consuming and not time-efficient.

In recent years, convolutional neural networks (CNNs) have developed rapidly in the fields of image classification, object detection and semantic segmentation, and have achieved great success in many fields, such as face recognition [8] and autonomous driving [9,10]. CNNs are mainly used for feature extraction through convolution operation to achieve the purpose of images classification or objection detection. Due to the fast speed of convolution operation, CNNs are very effective in dealing with large-scale datasets and under the drive of a certain scale of data, CNNs can achieve unexpected good results. Therefore, more and more algal researchers have begun to apply CNNs to the classification and detection of algae. Moreover, the acquisition and collection of algal images are difficult due to their obvious regional nature. Most algal researchers use data augmentation methods to expand the numbers of algal images [11,12,13,14]. These extended algal datasets can easily enable classification algorithms to achieve accuracy of more than 99% [11,13,15] and average precision of more than 80% [15,16], which leads to the classification and detection performance of CNN on algal dataset not being able to be well mined. Furthermore, most algal researchers focus on algal classification and there are few studies on algal detection. The reason is that the algal classification task requires the CNN to achieve the correct output of the category for the input image containing a single algal species, while the algal detection task requires the CNN to achieve the correct output of the category and location for the input image containing multiple algal species. Compared with algal classification, algal detection requires more complex dataset and algorithms [17]. The existing literature on algal detection not only uses a small dataset containing limited algal species, but also only proves that object detection algorithms can be applied to the algal detection task. In other words, the existing studies cannot realize the real-time algal detection and timely warning before algal blooms occur.

Therefore, based on the object detection algorithm, we construct an efficient self-organized detection system for algae. In order to better build and debug the algal self-organized detection system, we use an interactive method to generate the algal detection dataset containing 28,329 images, 562,512 bounding boxes and 54 genera. Based on this algal detection dataset, we explore and compare 10 different versions of state-of-the-art object detection algorithms. The experimental results show that under the same dataset and training conditions, YOLOv7 has the best detection results. We will consider replacing the YOLOv5 embedded in the algal self-organized detection system with YOLOv7. In practical application, the system not only has good algal detection results, but also can complete the scanning, photographing and detection of a 2 cm × 2 cm, 0.1 mL algal slide specimen within five minutes (resolution is 0.25886 μm/pixel); such a task requires a well-trained algal expert to work continuously for more than three hours.

At present, the collection and transmission of algal data depend on manual completion, which hinders the real-time detection of algae. The Internet of Things (IoT), which aims to enable ubiquitous wireless connections among various smart sensors, actuators and intelligent controllers and then integrate their functions to realize the mutual sharing and interaction of information, can be used to collect and transmit algal data in real-time. With the help of the IoT and the algal self-organized detection system built by us, real-time detection of algae can be well realized in the future. At the same time, we can upload the results to the cloud through the IoT, which is used as the basis for algal bloom warning. The contribution of this paper can be stated as follows:

- An algal self-organized detection system is established, which not only guarantees the good detection results, but also realizes the rapid detection of algae;
- An interactive method for generating an algal detection dataset is proposed;
- The detection performance of 10 different versions of state-of-the-art object detection algorithms is compared on the algal detection dataset.

The rest of this paper is as follows. Section 2 gives the related work. Section 3 introduces the research material and methods in detail. Experimental implementation and experimental results are presented in Section 4. Finally, we conclude the paper in Section 5.

## 2. Related Works

Object detection algorithm: As we know, object detection is one of the core research contents of computer vision. Its purpose is to predict the position of objects in a given image and label each object with a corresponding category. In the early stage, object detection is divided into three steps: generating region proposal, extracting features and region classification [18]. The most representative algorithm of this method is the deformable part-based model (DPM) algorithm [19] extended on histogram of oriented gradients (HOG) [20], which won the 2007, 2008, 2009 detection challenge on Pascal VOC dataset [21].

After CNN achieved a higher accuracy of image classification [22], a wave of research on object detection using CNN was set off. Currently, object detection based on deep learning has formed two factions: two-stage detection and one-stage detection. In the two-stage detection framework, the first stage generates the candidate region proposals and uses CNN for feature extraction. Then, the second stage uses a specific classifier to predict the categories of the candidate region proposals. The most representative algorithm is R-CNN [23] and its series of optimization deformation algorithms, such as Fast R-CNN [24], Faster-RCNN [25], Feature Pyramid Network (FPN) [26]. In the one-stage detection framework, all locations of the whole image are regarded as potential objects by default, the bounding boxes and categories of the objects are predicted simultaneously on the feature map. Its representative algorithm is You Only Look Once (YOLO). Since Joseph et al. [27] first proposed the YOLO algorithm in 2015, there have been seven versions of YOLO, namely YOLOv1-YOLOv7 [27,28,29,30,31,32,33].

CNN application in algal identification: Recently, with the remarkable success of CNN in various industries, a large number of algal researchers using CNN to identify algae have emerged. Pedraza et al. [11] first applied CNN to the classification of 80 diatoms, using the data augmentation approach to expand the dataset to over 160,000 samples and finally achieved an overall accuracy of 0.99 in AlexNet. Park et al. [12] used the neural architecture search (NAS) technology to design a CNN suitable for algal image classification and obtained an F1-score of 0.95 on eight algal genera. In addition, they also discussed the impact of data enhancement on classification. Several repeated experimental results show that the classification results after data enhancement are lower than those using the original dataset. Yadav et al. [13] used data augmentation techniques to expand the algal dataset 100 images to 80,000 images. Based on the expanded dataset of 16 algal families, ResNeXt was modified and a classification accuracy of 0.9997 was finally achieved. Xu et al. [14] expanded 13 algal species through data enhancement, forming a relatively balanced dataset among different algal species. Based on this dataset, they designed a new CNN algorithm, which obtained the lowest classification probability of 0.939.

While the above studies mainly focus on the classification of algae, the detection of algae has also attracted the attention of algal researchers. Samantaray et al. [34] proposed a viable algal monitoring system that uses transfer learning techniques to test three object detection algorithms, Faster R-CNN, Single Shot Detector (SSD) and Region-based Fully Convolutional Networks (R-FCN), on two datasets of hundreds of ground algal images and aerial algal images. The final monitoring system chooses the more robust, accurate and faster reasoning R-FCN algorithm. Baek et al. [16] used R-CNN and CNN to detect and count five cyanobacteria species and the average precision values of the final detection were between 0.89 and 0.929. Qian et al. [35] proposed a new object detection network based on faster R-CNN and tested the new network on the algal dataset containing 27 genera, achieving 0.7464 mean average precision (mAP). Park et al. [17] compared YOLOv3 with YOLOv4 on a dataset of 437 images containing 30 algal genera and showed that YOLOv4 performed better. Salido et al. [15] proposed a low-cost automated digital microscopy platform for the automatic classification and detection of diatoms. On a dataset containing 80 species of algae, they achieved a detection accuracy of 0.86 using YOLO and a classification accuracy of 0.9951 using AlexNet. Ali et al. [36] applied deep convolutional generative adversarial neural (DC-SGAN) to expand the dataset containing four types of algae and carried out comparative experiments on YOLOv3, YOLOv4 and YOLOv5 on the expanded dataset. The comparison results show that YOLOv5 has the best performance.

Different from the above studies, based on the object detection algorithm, we build the efficient algal self-organized detection system, which can automatically scan the algal slide specimens and realize the algal self-organized detection. The system is designed to achieve real-time detection of algae, so as to give a timely warning of algal blooms.

## 3. Material and Methods

In this section, we first introduce the built algal self-organized detection system. Then, we present the data acquisition and pre-processing, give the specific steps of interactive algal detection dataset generation method and show the detailed information of the algal detection dataset we have established. Finally, the evaluation standard of object detection is proposed.

### 3.1. Algal Self-Organized Detection System

**Figure 1:**
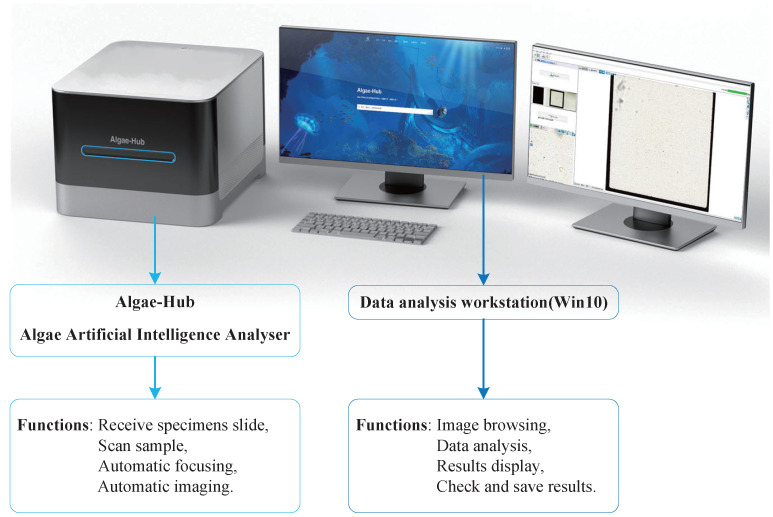
The demonstration picture of the algal self-organized detection system and the functions of its major components.

The algal self-organized detection system is mainly composed of the Algae-Hub Algae Artificial Intelligence Analyser (AH-20-S, Jiangsu Metabio Science & Technology Co., Ltd., Wuxi, China), data analysis workstation, display, mouse and keyboard. The system we built and the functions of its major components are shown in Figure 1.

The Algae-Hub consists of an objective lens and an imaging camera, accepting 20 mm × 20 mm, 0.1 mL algal specimens in a slide. The scan magnification of the Algae-Hub is 20× or 40×, the resolution is 0.25886 μm/pixel and the scanning speed is less than 120 s. It can realize automatic focus or manually fine-tune the focus. In addition, the embedded camera is a 5-megapixel CMOS camera.

The data analysis workstation is a computer installed with a win10 operating system, and also installed with Artificial Intelligence Analyser professional analysis software. It analyzes the images generated by the Algae-Hub independently. The methods of analyzing the images include the visual method, the diagonal method, the lattice method and the whole section method. By default, the entire image is evenly divided into 100 square grids. The visual method analyzes a selected square grid, the diagonal method analyzes the diagonal square grids of the 100 square grids, the lattice method selects the square grids of some rows for analysis and the whole section method analyzes the entire image. After the analysis is completed, the workstation displays the name and number of algal species identified in the selected square grids and saves the image detection results in the workstation. For the detection results, we can view, verify and modify. Once we have optimized the system, we just put in algal slide specimens or algal images, choose an analysis method and the system can realize self-organized algal detection. In addition, the workstation integrates functions such as statistical algal density and distribution. We can also continue to add features as needed in the future.

**Figure 2:**
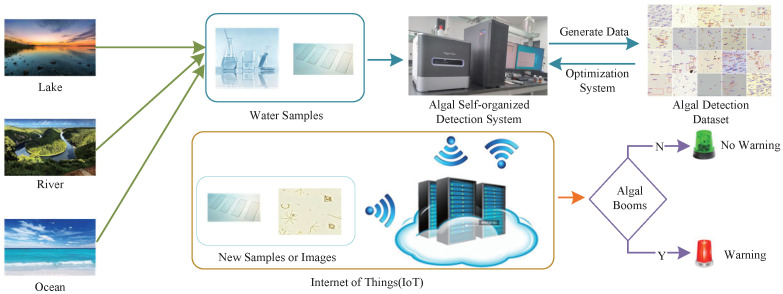
The schematic diagram of the algal self-organized detection system under the IoT.

In the context of the IoT, we focus on using various smart sensors, actuators and intelligent controllers to achieve real-time acquisition and sharing of algal data in the future. Combined with the efficient algal self-organized detection system we built, the algal self-organized detection system under the IoT is formed to implement the real-time detection of algae and the interaction of detection results, so as to realize a timely warning of algal blooms. In order to better display the specific workflow of the algal self-organized detection system under the IoT in the future, we present the schematic diagram in Figure 2.

It is worth noting that the core of the detection system is the object detection algorithm integrated in the system. As we know, although the two-stage detection method has good detection performance, the detection speed is far slower than that of the one-stage detection method. Therefore, we chose the YOLO series in the one-stage detection method to accomplish the task of real-time object detection. While there are many object detection algorithms in the YOLO series, YOLOv5 is one of the most stable and widely used. Initially, the algal self-organized detection system we built was based on YOLOv5, whose performance meets our requirements.

### 3.2. Dataset Acquisition and Pre-Processing

We collect water samples through several pilot sites in Taihu Lake in Wuxi, China. For the water samples with algal cells gathered together, the algal cells are scattered as much as possible by shaking or ultrasonic crushing, while for the water samples with large density of algal cells, appropriate dilution is carried out.

The processed water samples are made into slide specimens and the algal images are automatically scanned and saved using our proposed algal self-organized detection system. It is worth noting that the algal detection dataset built by us is generated interactively and the specific steps are as follows:

(i) For the slide specimens, the algal self-organized detection system automatically scans to obtain algal images.
(ii) The algal images acquired in (i) are cross-labeled by 15 algal experts using labelImg annotation software with reference to the VOC dataset format and the annotation files are saved. Then, the initial algal detection dataset is generated.
(iii) The algal detection dataset generated in (ii) is trained by the object detection algorithm to obtain the optimal weight and the optimal weight is imported into the algal self-organized detection system.
(iv) For the new slide specimens or images, the algal self-organized detection system implements automatic scanning, analysis and export of the analysis result images.
(v) The algal images analyzed in (iv) are manually checked and combine with the algal detection data produced in (ii) to generate a new algal detection dataset.
(vi) Repeat (iii)–(v) to finally obtain the algal detection dataset used in this paper.

Among the above steps to interactively generate the algal dataset, the dataset in (ii) is used to start and tune the algal self-organized detection system we have built. New samples are detected in (iv) to expand and enrich the algal detection dataset. The manual check in (v) is to eliminate the detection errors produced by the algal self-organized detection system. Through interactive data generation, we can quickly obtain a considerable number of algal detection datasets with relatively accurate labels and bounding boxes. Based on this dataset, we can adjust the algal self-organized detection system to the optimal state, so as to detect algae more quickly and accurately.

For our algal dataset, each image has a width and height of pixels between 1536 and 1984. Due to the inconsistent number of bounding boxes for various algae, we select algae with more than 200 bounding boxes as the final algal detection dataset. Therefore, we obtain an algal detection dataset consisting of 28,329 images, 562,512 bounding boxes and 54 genera. We randomly split the dataset into three parts, training set, validation set and test set and their ratio is 7:2:1. The specific information of the datasets is presented in Table 1.

**Figure 3:**
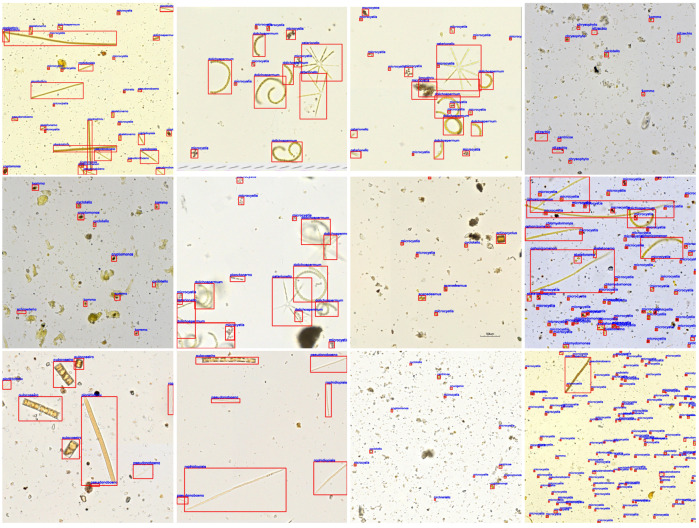
Twelve algal detection images with annotation information. Images are randomly selected from the algal detection dataset generated via the interactive method. Each image corresponds to an annotation file, which stores the location information and the category of algae. We use red rectangles and blue fonts to display the bounding boxes and categories on the chosen images.

As shown in Table 1, we give the names of 54 algae that reach the genus level, the number of images for each algal species and the number of bounding boxes for each algal species after data division. Note that there are multiple algal genera in an image, the sum of the images of each algal genus is not the total number of algal images. To better display the algal detection dataset constructed in this paper, we randomly select 12 images, draw their bounding boxes and categories and show them in Figure 3.

### 3.3. Detection Evaluation

As we know, the object detection goal is to find the location of the object in the image and give the corresponding label. For the predicted object locations, we use intersection over union (IOU) to evaluate the accuracy, which is calculated by the following formula:

$$\mathrm{IOU}=\frac{Area\left(b_{pred}\right)⋂Area\left(b_{truth}\right)}{Area\left(b_{pred}\right)⋃Area\left(b_{truth}\right)},$$

where $Area(b_{pred})$ represents the area of the predicted bounding box and $Area(b_{truth})$ presents the area of the ground truth bounding box [21]. When the value of the IOU between the predicted bounding box and the ground truth bounding box is greater than the preset IOU threshold, the location detection is correct. Otherwise, it is regarded as missed detection.

For the predicted labels, average precision (AP) is used for evaluation. AP is the area under the precision–recall curve calculated by interpolation. The precision (P) and recall (R) are calculated as follows:

$$P=\frac{\mathrm{TP}}{\mathrm{TP}+\mathrm{FP}},\;R=\frac{\mathrm{TP}}{\mathrm{TP}+\mathrm{FN}},$$

where TP is true positive, representing the number of true positive samples that are correctly predicted. FP is false positive, representing the number of true positive samples that are incorrectly predicted. FN is false negative, representing the number of true negative samples that are incorrectly predicted.

In image classification, positive samples refer to the samples of the current category, while negative samples refer to the remaining samples that do not belong to the current category. In object detection, the definition of positive and negative samples is more complex and even varies in different periods. For example, YOLOv3 [29] uses the dual IOU thresholds strategy; that is, the thresholds are 0.3 and 0.7 respectively. For a predicted bounding box, if its IOU with the ground truth bounding box is greater than 0.7, it is considered as a positive sample; if its IOU with the ground truth bounding box is between 0.3 and 0.7, it is ignored. If its IOU with the ground truth bounding box is less than 0.3, it is considered as a negative sample. YOLOv4 [30] states that for a predicted bounding box, if its IOU with the ground truth bounding box is greater than the preset threshold, it is a positive sample. Otherwise, it is a negative sample. In general, we use the mean AP (mAP) of the average over all objects as the indicator to evaluate the prediction label.

In order to take into account both the evaluation of the predicted location and label, mAP with an IOU of 0.5 (mAP@.5) and an average mAP of 10 different IOU thresholds with a step size of 0.05 between 0.5 and 0.95 (mAP@.5:.95) is commonly used to evaluate the performance of the object detection algorithm. Under the same dataset and training environment, the larger the mAP@.5 and mAP@.5:.95 values, the better the detection performance of the object detection algorithm.

## 4. Experiments

Recently, YOLOv6 and YOLOv7 appeared, which performs better than YOLOv5 on the COCO dataset. Therefore, this section gives the parameters and equipment for experiment implementation, as well as the detection results of 10 different versions of YOLOv5, YOLOv6 and YOLOv7 on the algal detection dataset.

### 4.1. Implementation

The experiments in this paper are based on pytorch 1.12, running on an ubuntu operating system with Intel(R) Core(TM) i9-12900k, a 3090 24GB GPU. Before providing the data to the network, we only convert the annotations to the format that YOLO needs. We use the default hyperparameter settings of YOLOv5, YOLOv6 and YOLOv7. We set the image size to 640×640 pixels and the batch size to 24. Each model is trained from scratch for 300 epochs and does not use pre-trained weights. When testing the training model, the confidence threshold is set to 0.001 for YOLOv5 and YOLOv7 and 0.03 for YOLOv6. All the IOU thresholds are set to 0.6.

### 4.2. Detection Results

Based on the algal detection dataset we built, 10 different versions of YOLOv5, YOLOv6 and YOLOv7 are trained on the training set and verified on the validation set, respectively. After the training, the parameter model with the best performance on the validation set is selected for the final test on the test set. Table 2 shows the detection results.

**Figure 4:**
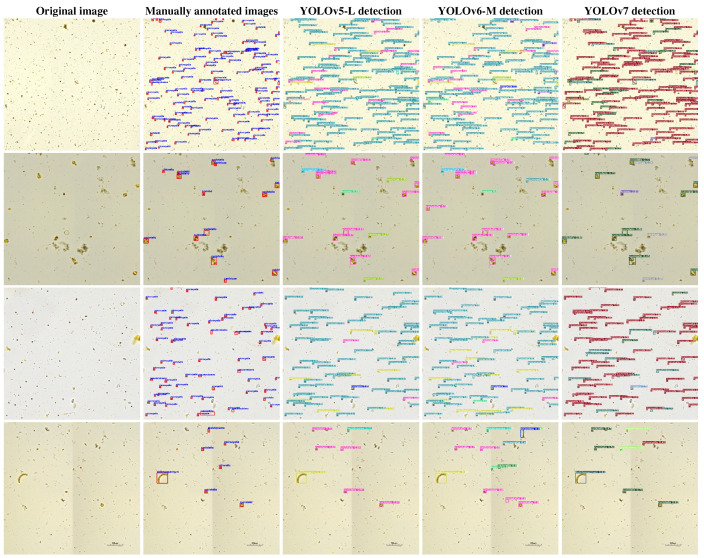
Schematic of four randomly selected images in the test set and the corresponding detection results. The four images in each row from left to right are the original image, the corresponding manually annotated image and the final detection results of YOLOv5-L, YOLOv6-M and YOLOv7.

It can be seen from Table 2 that under the same input condition of 640×640 pixels, under the same detection model framework, the larger the model, the higher the mAP@.5 and mAP@.5:.95 and the smaller the frames per second (FPS). Hence, YOLOv5-L, YOLOv6-M and YOLOv7 achieve the highest mAP@.5 and mAP@.5:.95 with their respective frameworks. Among 10 different versions of YOLOv5, YOLOv6 and YOLOv7, YOLOv7 has the highest mAP@.5, but YOLOv5 has the highest mAP@.5:.95. Compared with YOLOv5-L and YOLOv6-M, YOLOv7 has the fastest frames per second (FPS). Therefore, we are considering replacing the YOLOv5-L algorithm embedded in the algal self-organized detection system with YOLOv7. Moreover, from Table 2, we can see that the detection speed (FPS) of 10 object detection algorithms is far higher than that of manual algal detection, which can well realize real-time algal detection. In order to better present the detection effect of the trained model in the test set, we set the confidence threshold to 0.25 and the IOU threshold to 0.45. Then, we randomly select four images and present them together with the corresponding manually annotated image, detection results of YOLOv5-L, YOLOv6-M and YOLOv7 in Figure 4.

From Figure 4, YOLOv5-L, YOLOv6-M and YOLOv7 can detect the algae that are not manually labeled although there are few missed algae. This fully proves that the object detection algorithm can surpass the manual algal detection method in algal detection. Meanwhile, Table 2 and Figure 4 also fully demonstrate the high efficiency of the algal self-organized detection system built by us based on YOLOv5.

## 5. Conclusions

In this paper, we first present the algal self-organized detection system we built. Then, we used an interactive method to generate an algal detection dataset containing 28,329 images, 562,512 bounding boxes and 54 genera. Based on the algal detection dataset, 10 different versions of YOLOv5, YOLOv6 and YOLOv7 were compared. The experimental results show that under the same dataset and training conditions, the detection performance of YOLOv7 is better than that of YOLOv5-L and YOLOv6-M. We will consider using YOLOv7 to replace YOLOv5-L embedded in the algal self-organized detection system. Meanwhile, in practical applications, the efficient algal self-organized detection system embedded with YOLOv5-L can realize rapid and accurate detection of algae. However, the current system relies on manual data acquisition and transmission, which hinders real-time detection of algae. In the future, we will focus on using intelligent sensors to realize real-time collection and sharing of algal data and combine with the algal self-organized detection system we have built to form an algal self-organized detection system under the IoT, so as to realize timely warning of algal blooms. This is one of our future major works.

## Figures and Tables

**Table 1:** Statistics of the algal detection dataset.

Categories	Train		Val		Trainval		Test	
	Images	Objects	Images	Objects	Images	Objects	Images	Objects
achnanthidium	556	915	137	220	693	1135	87	138
actinocyclus	566	755	156	220	722	975	85	111
aphanizomenon	756	1344	211	433	967	1777	121	216
aphanocapsa	264	308	68	73	332	381	40	48
asterionella	356	637	105	172	461	809	54	101
aulacoseira	3126	3911	874	1089	4000	5000	418	533
centricae	1951	4693	590	1421	2541	6114	256	608
chlamydomonas	1234	1667	373	487	1607	2154	189	239
chlorella	3334	6995	944	1910	4278	8905	487	990
chlorophyta	141	164	37	46	178	210	21	23
chromulina	199	275	64	77	263	352	31	41
chrysophyta	819	1285	241	419	1060	1704	109	170
coelastrum	172	180	50	50	222	230	20	22
cosmarium	426	521	106	132	532	653	75	101
crucigenia	608	676	187	207	795	883	78	91
cryptomonas	2932	4335	826	1211	3758	5546	405	598
cryptophyta	204	638	59	159	263	797	33	116
cuspidothrix	557	858	155	249	712	1107	103	147
cyanophyta	84	288	17	45	101	333	7	26
cyclotella	8262	27,625	2408	8035	10,670	35,660	1210	4052
cylindrospermopsis	369	2328	108	640	477	2968	44	242
desmodesmus	890	959	274	306	1164	1265	124	133
dinobryon	163	166	32	33	195	199	22	22
dinophyta	180	187	53	55	233	242	30	32
dolichospermum	2832	8783	789	2746	3621	11,529	367	776
euglena	134	146	42	45	176	191	17	19
kirchneriella	1086	1268	368	426	1454	1694	152	176
komma	4664	16,155	1429	4878	6093	21,033	667	2425
limnothrix	1581	3700	430	1021	2011	4721	248	545
merismopedia	422	546	125	154	547	700	64	85
microcystis	7316	244,604	2164	73,860	9480	318,464	1019	32,796
monoraphidium	438	464	130	145	568	609	61	66
mougeotia	574	1111	142	270	716	1381	90	201
navicula	410	570	100	132	510	702	56	70
nitzschia	1991	2682	560	759	2551	3441	258	345
oocystis	1710	2145	480	609	2190	2754	259	324
pediastrum	172	174	42	42	214	216	23	25
pennatae	200	256	48	65	248	321	36	40
peridiniopsis	93	146	22	38	115	184	16	31
phacotus	127	168	36	55	163	223	19	32
planctonema	493	815	144	229	637	1044	57	95
planktosphaeria	1156	1923	331	499	1487	2422	171	300
planktothricoides	1042	2155	270	540	1312	2695	142	284
planktothrix	1597	3508	463	992	2060	4500	231	536
pseudanabaena	4281	14,506	1227	4139	5508	18,645	625	2058
raphidiopisis	1212	2191	345	635	1557	2826	169	311
rhabdogloea	1086	1358	278	358	1364	1716	141	181
scenedesmus	1706	1949	498	557	2204	2506	249	288
schroederia	332	348	81	85	413	433	46	50
skeletonema	3561	15,738	1017	4513	4578	20,251	466	2033
tetradesmus	150	171	43	50	193	221	24	25
tetraedron	739	834	194	221	933	1055	107	117
trachelomonas	274	287	90	100	364	387	45	52
ulnaria	1766	2215	511	612	2277	2827	261	336
Total	19831	392,626	5665	116,464	25,496	509,090	2833	53,422

**Table 2:** Comparison of detection results on algal dataset.

Model	Parameters	FLOPs	mAP@.5	mAP@.5:.95	FPS
YOLOv5-N (r6.2) [31]	1.8M	4.4G	56.1%	38.0%	435
YOLOv5-S (r6.2) [31]	7.2M	16.2G	65.9%	45.7%	370
YOLOv5-M (r6.2) [31]	21.1M	48.5G	68.3%	49.0%	208
YOLOv5-L (r6.2) [31]	46.4M	108.6G	69.6%	**50.5%**	114
YOLOv6-N [32]	4.31M	11.1G	56.5%	39.4%	658
YOLOv6-T [32]	9.69M	24.88G	61.1%	43.0%	383
YOLOv6-S [32]	17.21M	44.14G	65.4%	46.2%	299
YOLOv6-M [32]	34.27M	82.12G	68.6%	50.1%	179
YOLOv7-Tiny [33]	6.2M	13.5G	61.7%	42.9%	526
YOLOv7 [33]	36.8M	104.1G	**70.6%**	50.4%	204

## Data Availability

Due to non-disclosure agreements, the dataset in this paper are not publicly available. The dataset can be made available to real algal researchers, but only if they sign non-disclosure agreements.

## References

[1] Harvell C.D., Kim K., Burkholder J., Colwell R.R., Epstein P.R., Grimes D.J., Hofmann E.E., Lipp E., Osterhaus A., Overstreet R.M. (1999). Emerging marine diseases–climate links and anthropogenic factors. Science.

[2] Van Dolah F.M. (2000). Marine algal toxins: Origins, health effects and their increased occurrence. Environ. Health Perspect..

[3] Landsberg J.H. (2002). The effects of harmful algal blooms on aquatic organisms. Rev. Fish. Sci..

[4] Davis C.C. (1948). *Gymnodinium brevis* sp. nov., a cause of discolored water and animal mortality in the Gulf of Mexico. Bot. Gaz..

[5] Heil C.A., Steidinger K.A. (2009). Monitoring, management and mitigation of Karenia blooms in the eastern Gulf of Mexico. Harmful Algae.

[6] Qin B., Zhu G., Gao G., Zhang Y., Li W., Paerl H.W., Carmichael W.W. (2010). A drinking water crisis in Lake Taihu, China: Linkage to climatic variability and lake management. Environ. Manag..

[7] Paerl H.W., Gardner W.S., McCarthy M.J., Peierls B.L., Wilhelm S.W. (2014). Algal blooms: Noteworthy nitrogen. Science.

[8] Sun Y., Liang D., Wang X., Tang X. (2015). Deepid3: Face recognition with very deep neural networks. arXiv.

[9] Li B., Ouyang W., Sheng L., Zeng X., Wang X. Gs3d: An efficient 3d object detection framework for autonomous driving. Proceedings of the IEEE/CVF Conference on Computer Vision and Pattern Recognition.

[10] Feng D., Haase-Schütz C., Rosenbaum L., Hertlein H., Glaeser C., Timm F., Wiesbeck W., Dietmayer K. (2020). Deep multi-modal object detection and semantic segmentation for autonomous driving: Datasets, methods and challenges. IEEE Trans. Intell. Transp. Syst..

[11] Pedraza A., Bueno G., Deniz O., Cristóbal G., Blanco S., Borrego-Ramos M. (2017). Automated diatom classification (Part B): A deep learning approach. Appl. Sci..

[12] Park J., Lee H., Park C.Y., Hasan S., Heo T.Y., Lee W.H. (2019). Algal morphological identification in watersheds for drinking water supply using neural architecture search for convolutional neural network. Water.

[13] Yadav D., Jalal A., Garlapati D., Hossain K., Goyal A., Pant G. (2020). Deep learning-based ResNeXt model in phycological studies for future. Algal Res..

[14] Xu L., Xu L., Chen Y., Zhang Y., Yang J. (2022). Accurate Classification of Algae Using Deep Convolutional Neural Network with a Small Database. ACS ES&T Water.

[15] Salido J., Sánchez C., Ruiz-Santaquiteria J., Cristóbal G., Blanco S., Bueno G. (2020). A low-cost automated digital microscopy platform for automatic identification of diatoms. Appl. Sci..

[16] Baek S.S., Pyo J., Pachepsky Y., Park Y., Ligaray M., Ahn C.Y., Kim Y.H., Chun J.A., Cho K.H. (2020). Identification and enumeration of cyanobacteria species using a deep neural network. Ecol. Indic..

[17] Park J., Baek J., Kim J., You K., Kim K. (2022). Deep Learning-Based Algal Detection Model Development Considering Field Application. Water.

[18] Wu X., Sahoo D., Hoi S.C. (2020). Recent advances in deep learning for object detection. Neurocomputing.

[19] Felzenszwalb P.F., Girshick R.B., McAllester D., Ramanan D. (2010). Object detection with discriminatively trained part-based models. IEEE Trans. Pattern Anal. Mach. Intell..

[20] Dalal N., Triggs B. Histograms of oriented gradients for human detection. Proceedings of the 2005 IEEE Computer Society Conference on Computer Vision and Pattern Recognition (CVPR’05).

[21] Everingham M., Van Gool L., Williams C.K., Winn J., Zisserman A. (2010). The pascal visual object classes (voc) challenge. Int. J. Comput. Vis..

[22] Krizhevsky A., Sutskever I., Hinton G.E. (2017). Imagenet classification with deep convolutional neural networks. Commun. ACM.

[23] Girshick R., Donahue J., Darrell T., Malik J. Rich feature hierarchies for accurate object detection and semantic segmentation. Proceedings of the IEEE Conference on Computer Vision and Pattern Recognition.

[24] Girshick R. Fast r-cnn. Proceedings of the IEEE International Conference on Computer Vision.

[25] Ren S., He K., Girshick R., Sun J. (2015). Faster r-cnn: Towards real-time object detection with region proposal networks. Adv. Neural Inf. Process. Syst..

[26] Lin T.Y., Dollár P., Girshick R., He K., Hariharan B., Belongie S. Feature pyramid networks for object detection. Proceedings of the IEEE Conference on Computer Vision and Pattern Recognition.

[27] Redmon J., Divvala S., Girshick R., Farhadi A. You only look once: Unified, real-time object detection. Proceedings of the IEEE Conference on Computer Vision and Pattern Recognition.

[28] Redmon J., Farhadi A. YOLO9000: Better, faster, stronger. Proceedings of the IEEE Conference on Computer Vision and Pattern Recognition.

[29] Redmon J., Farhadi A. (2018). Yolov3: An incremental improvement. arXiv.

[30] Bochkovskiy A., Wang C.Y., Liao H.Y.M. (2020). Yolov4: Optimal speed and accuracy of object detection. arXiv.

[31] Jocher G., Chaurasia A., Stoken A., Borovec J., Kwon Y., Michael K., Fang J. (2022). ultralytics/yolov5: v6.2-YOLOv5 Classification Models, Apple M1, Reproducibility, ClearML and Deci.ai integrations. GitHub.

[32] Li C., Li L., Jiang H., Weng K., Geng Y., Li L., Ke Z., Li Q., Cheng M., Nie W. (2022). YOLOv6: A single-stage object detection framework for industrial applications. arXiv.

[33] Wang C.Y., Bochkovskiy A., Liao H.Y.M. (2022). YOLOv7: Trainable bag-of-freebies sets new state-of-the-art for real-time object detectors. arXiv.

[34] Samantaray A., Yang B., Dietz J.E., Min B.C. (2018). Algae detection using computer vision and deep learning. arXiv.

[35] Qian P., Zhao Z., Liu H., Wang Y., Peng Y., Hu S., Zhang J., Deng Y., Zeng Z. Multi-target deep learning for algal detection and classification. Proceedings of the 2020 42nd Annual International Conference of the IEEE Engineering in Medicine & Biology Society (EMBC).

[36] Ali S., Khan Z., Hussain A., Athar A., Kim H.C. (2022). Computer Vision Based Deep Learning Approach for the Detection and Classification of Algae Species Using Microscopic Images. Water.

